# Impact of postural variation on hand measurements: Three-dimensional anatomical analysis

**DOI:** 10.1371/journal.pone.0250428

**Published:** 2021-04-23

**Authors:** Mei-ying Kwan, Kit-lun Yick, Lung Chow, Annie Yu, Sun-pui Ng, Joanne Yip

**Affiliations:** 1 Institute of Textiles and Clothing, The Hong Kong Polytechnic University, Kowloon City, Hong Kong; 2 Department of Advanced Fibro Science, Kyoto Institute of Technology, Kyoto, Japan; 3 College of Professional and Continuing Education, The Hong Kong Polytechnic University, Kowloon City, Hong Kong; National Tsing Hua University, TAIWAN

## Abstract

In this article, the impact of postural variations on hand anthropometry and distribution of skin strain ratios has been investigated. The literature suggests the glove fit directly affects hand functions. However, gloves currently manufactured based on a static posture failed to provide optimum fit. Workers often do not wear protective gloves due to discomfort caused by improper design, which increases the risk of hand injury. Full-color three-dimensional scans of the hands are captured with thirty healthy subjects (20 females, 10 males) to analyze the hand measurements and skin deformation with various postures. 42 of the 57 hand dimensions were found to have significant differences (p >0.05) related to hand posture. The skin strain ratios further suggest that the slant of the web space, dorsal-length and surface area should be increased, while the angles of the web space and length of the palm reduced to advance glove patterns. This research contributes to constructing gloves with optimum fit, performance, and comfort. Results show that in consideration of hand postures, the angle of the slant of web space between digits 2 and 5 and the finger length on the dorsal side should be increased, whilst the finger length on the palm side should be reduced in glove pattern design. Gloves currently constructed based on a splayed posture cannot provide a good fit. Consideration should be given to hand measurements in dynamic postures.

## Introduction

### The glove fit problems

Gloves are commonly worn during work and sporting activities for protecting the hands, thus allowing better grasp and dexterity, and even increasing the safety of the task that is being carried out. Nevertheless, the complex anatomical structure of the hand means that gloves are one of the most complicated engineered products. To allow wearers to move their hand freely and flexibly, gloves must fit like a second skin to wearers for optimal comfort and practical use.

Poor-fitting gloves not only negatively affect the hand performance of the wearers, but also lead to continuous inefficient use or overuse of the hand muscles. It is inevitable that workers in many industries frequently do not wear protective gloves due to discomfort caused by an inappropriate design, which increases the risk of hand injuries [1]. This is also a typical problem with firefighters and astronauts and results in substantial strength degradation, reduced hand dexterity and increased risk of exposure to burns and other environmental hazards when they work barehanded [2–6].

To improve glove fit, hand anthropometry research has attempted to improve glove fitting and their association with hand performance. Yu et al. [7] indicated that glove fit in terms of the finger length dimensions is significantly correlated with the force of gripping, whilst the glove fit in terms of the hand, wrist and finger circumferences have a significant impact on the ability to handle small objects. A positive correlation between pinch strength and anthropometric measures which include the hand length, hand width, hand span, mid-arm and forearm circumferences have been reported in Mohammadian et al. [8].

### Skin deformation of the hand and finger web at hand movement

Nevertheless, the way that the hand is measured is important for improving glove fit. Since the hand dimensions change during movement, more detailed ergonomic and anthropometric analyses of the hand postures are crucial. Previous research on hand anthropometry has primarily focused on the hand dimensions in a splayed posture (static position). The changes in the hand dimensions with various hand postures have been largely neglected. By using three-dimensional (3D) scanning technology, few research studies have started to carry out dynamic measurements of the hand surface. Nasir and Troynikov [9] found that hand postures cause substantial changes in the geometry and curvature of the dorsal hand, especially in the metacarpal areas. The findings are in line with the significant changes in body measurements that correspond to body movement at the joints, which need to be measured in dynamic postures [10, 11]. Griffin et al. [12] further examined the changes in hand measurements relative to posture. They measured and compared the total length of the gripped hand with that of a flat hand. The outcome of their investigation showed that the total length on the dorsal side of the hand on average increased by 5%. Therefore, it was concluded that such variations in hand dimensions and skin deformation may result in significant changes to the strain distribution placed on the glove fabric as a result of the hand postures. The lack of information on dynamic hand postures in glove design may therefore lead to wear discomfort and limit the free movement of the hand and fingers [3]. It is anticipated that the quantification of skin strain with appropriate ease allowance in glove design and development would enable dynamic freedom of movement while avoiding undesired pressure that could impede the hand and finger performances and ranges of motion. Previous research, however, only focused on the back of the hand, regardless of the palm side or web space area. There is still much ambiguity in the evaluation of hand measurement changes with postures, leading to inconclusive results and significant challenges in advancing glove fit.

Another important aspect of hand anthropometry that affects the fit of gloves is the geometrical consideration of the 3D concave web space between the fingers. Yu et al. [7] introduced a new approach that takes the slant of the finger webs into account in the construction process of glove patterns to improve the fit of pressure therapy gloves. They explored the slant angle of the web space with 79 individuals by using 3D scanning. They found that glove prototypes that take into consideration the slant of the finger webs offer a better fitting, comfort and ease of hand motion. It is believed that the substantial geometry changes in the metacarpal areas which are contingent on finger movement may inevitably result in dimensions and curvature changes of the web space between the fingers during various activities in daily living. Nevertheless, specific information on the web space between the fingers and the corresponding changes to hand postures is largely absent.

To the best of our knowledge, no studies have been conducted on the effects of palm measurements and the distribution of skin strain ratios in relation to different hand postures. Measurements of the palm side of the hand should be equally important in fabricating a fitted glove, which also affects hand performance and wear comfort. This study therefore aims to analyze the hand anthropometry with a focus on hand postures, the variations in the skin deformation between the pronated dorsal and supinated palm sides of the hand with different hand postures, as well as the slant of the web spaces to improve the gloves fit, comfort, performance and functionality. In understanding the location and direction of the maximal stretching and posture of the hand, the fingers and web slants therefore provide glove designers with insight into the material properties and glove geometries that are necessary for optimum fit and protection.

## Materials and methods

### Subjects

A total of thirty healthy subjects (including 20 females and 10 males) were recruited at universities through electronic messages. The selected subjects: (1) were between the ages of 18 and 40 years old, and (2) had no history of hand injuries. The demographics of the participants are listed in Table 1. The percentage of glove sizes in the sample population is determined based on the glove size system shown in the reference [13]. The study was approved by the Human Ethics Committee of the Hong Kong Polytechnic University (Reference Number: HSEARS2017214001). The participants were informed and provided written consent before taking part in the study. The individual in this manuscript has given written informed consent (as outlined in PLOS consent form) to publish these case details. The study was in accordance with the latest revision of the Declaration of Helsinki [14].

**Table 1:** Participant demographics.

	Female N = 20	Male N = 10	Overall N = 30	Range N = 30
	(Mean ± S. D.)	(Mean ± S. D.)	(Mean ± S. D.)	
**Age (Years)**	26.55 ± 4.36	28.50 ± 4.74	27.20 ± 4.51	18.00–38.00
**Body height (cm)**	160.43 ± 3.86 *	171.60 ± 6.28 *	164.15 ± 7.12	154.00–180.00
**Body weight (kg)**	53.45 ± 6.36	67.45 ± 9.06	58.11 ± 9.85	44.00–90.00
**BMI (kg/m**^**2**^**)**	20.73 ± 1.91	22.85 ± 2.09	21.44 ± 2.19	18.55–27.78
**Hand circumference (mm)**	183.50 ± 8.50	207.10 ± 8.40	191.40 ± 14.00	229.50–164.40
**Hand length (mm)**	182.50 ± 6.60	199.50 ± 9.40	188.10 ± 11.10	213.90–171.10
**% of glove size (Small/Medium)**	95%/5%	90%/10%		

*Remarks: meet the 50^th^ percentile body height of Chinese.

### Experimental procedure

#### 3D scanning approach

3D scanning method was used to obtain the hand dimension measurement at 3 different hand postures. 3D scanners can accurately capture body shape and size, and even detailed hand information [15–19]. Nasir and Troynikov [9], Nasir, Troynikov and Watson [20] and Griffin et al. [12] used 3D scanning systems in greyscale to measure the hand dimensions and skin deformation. A full-color Artec Eva handheld 3D scanner (Artec 3D, Luxembourg) was used in this study to capture 3D images of the hands. The reliability, accuracy and reproducibility of this scanner have been validated in previous studies [16, 21, 22]. The Artec Eva 3D scanner has an accuracy of up to 0.1 mm and a resolution of up to 0.5 mm. The scanned images were processed by using Artec Studio 13 Professional software (Artec 3D, Luxembourg).

#### Experimental setup

**Fig 1:**
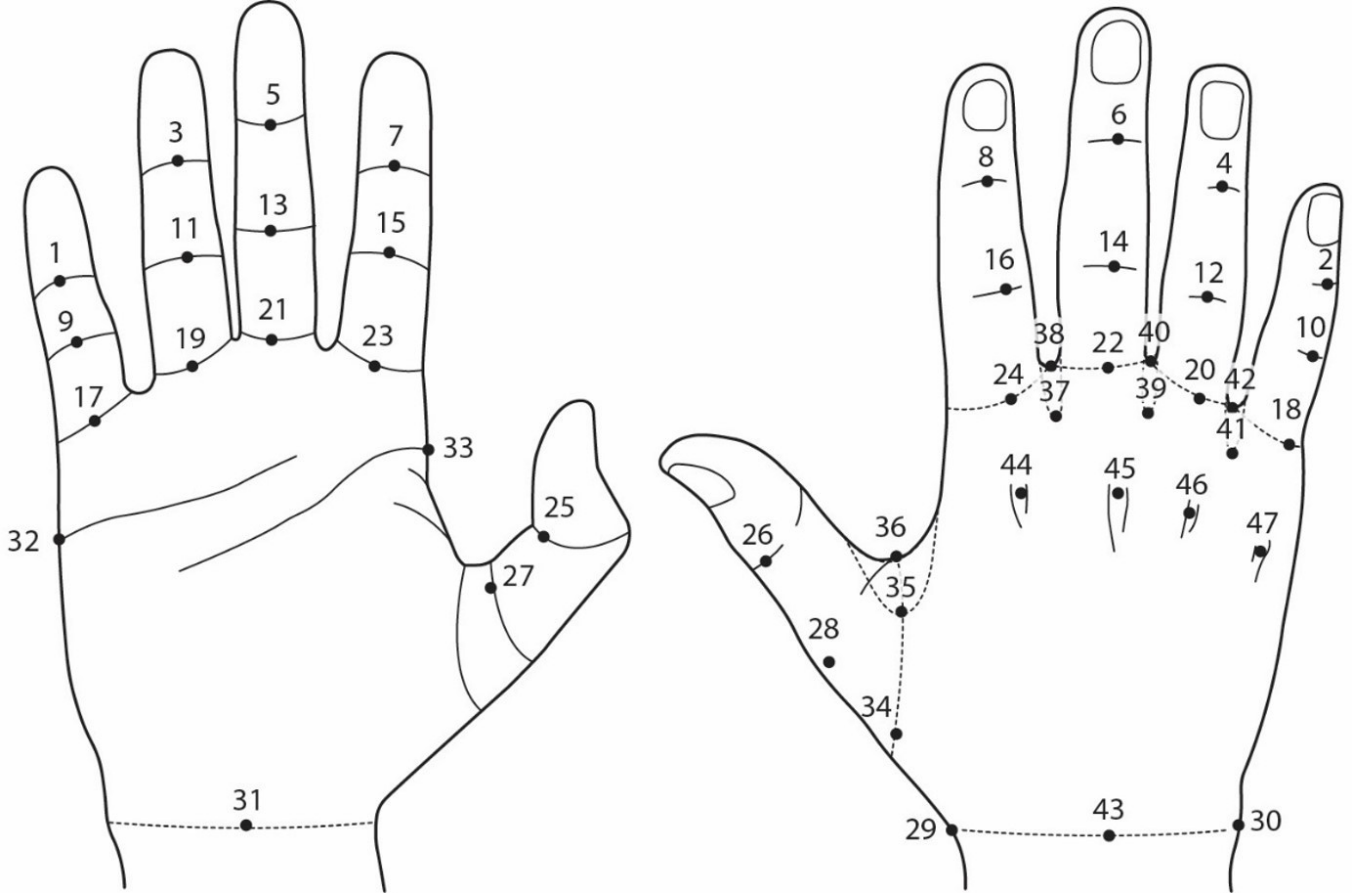
Landmarks of hand.

**Fig 2:**
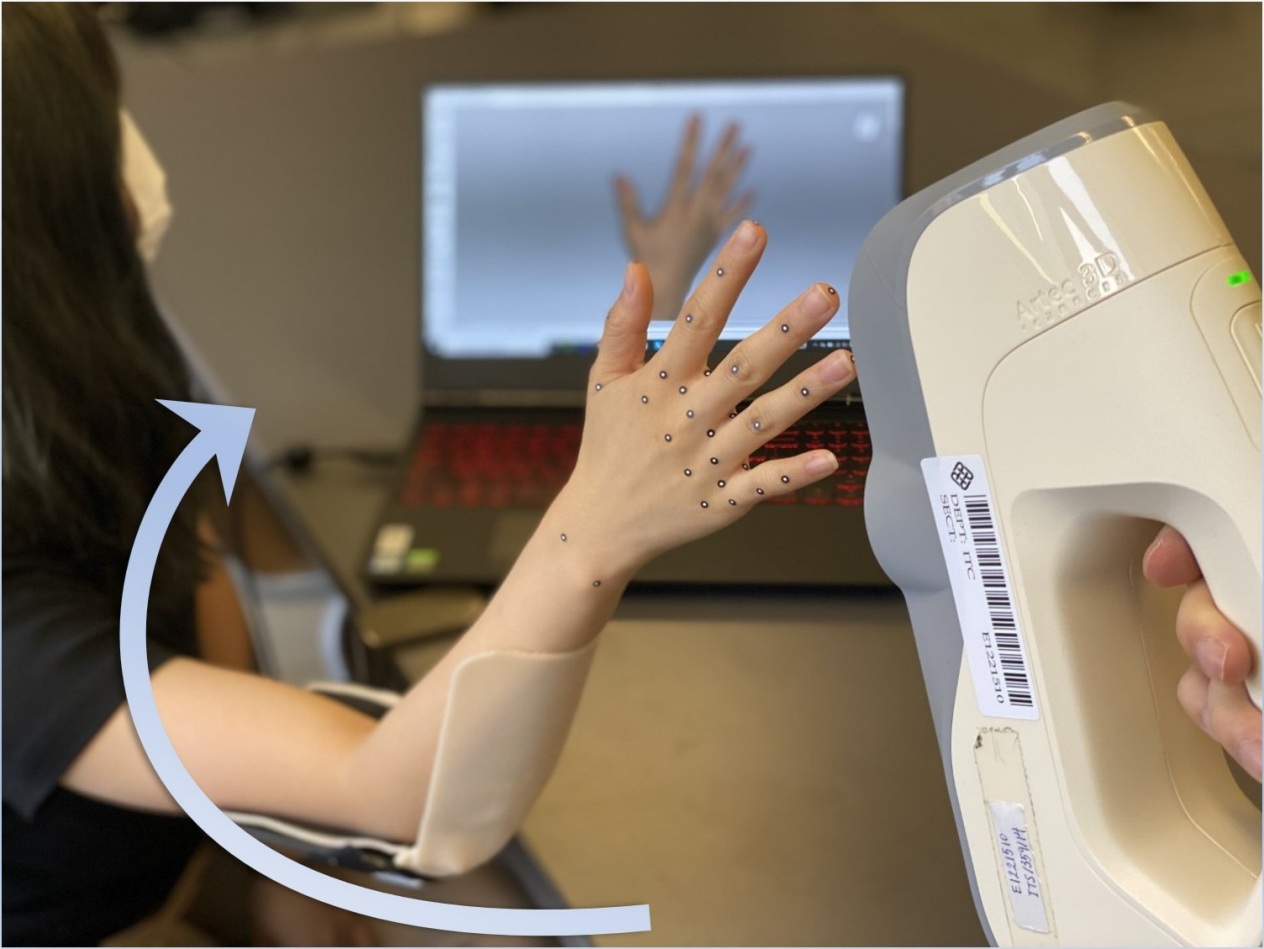
3D scanning of a splayed hand posture.

Prior to scanning, 47 colored pieces of landmarks that are 3 mm in diameter were accurately adhered to the hands of each subject to ensure reliable measurements for each posture, as shown in Fig 1. The use of small flat markers and full color scanned images can minimize the measurement errors with good data accuracy. The scanning process requires 30 seconds for each scan. Participants were scanned in bare hand condition. Since human subjects cannot maintain the same posture for a long period of time during scanning, a device was designed to provide suitable support to their elbows. The operator needs to carry the scanner around the subject’s hand to capture the image. Fig 2 shows scanning of a splayed hand posture. In this study, **a**n experienced operator was assigned to conduct the scanning, landmark adhesion, and image measurement to ensure consistent quality. The scanning software can identify subtle changes in the hands during the scanning process in time. Therefore, the operator can immediately obtain the best inspection of the 3D hand image. Each subject was repeatedly scanned for 2 to 4 times at each posture condition that at least two successful and complete 3D hand images can be obtained.

#### Hand measurements

**Fig 3:**
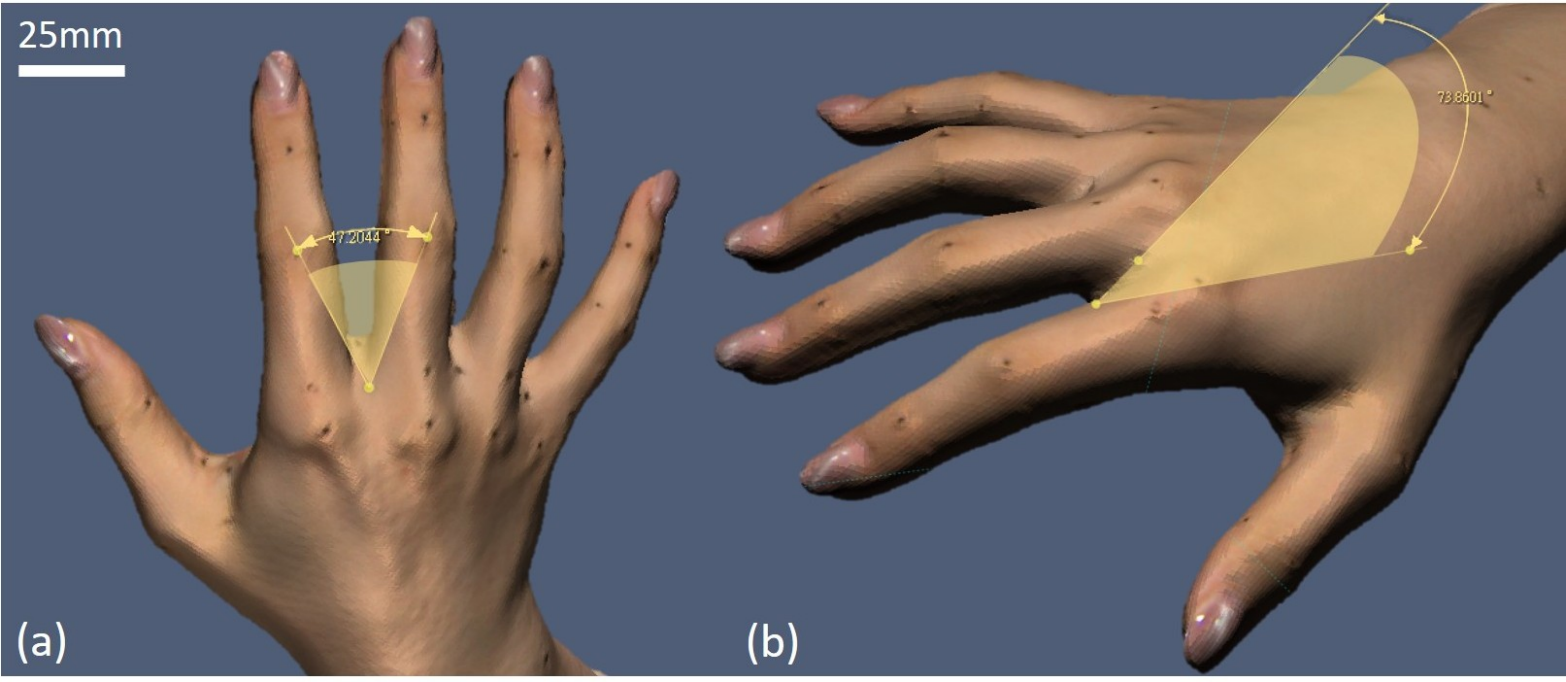
(a) Web space angle between D2 and D3, and (b) slant of web space between D2 and D3 measured in Rapidform XOR 3D scanning software.

The dimensions were measured with Rapidform XOR 3D scanning software (3D Systems Corporation, California, United States). There are 57 measurements, including 17 circumferential dimensions, 30 length dimensions, 8 angular dimensions and 2 surface area dimensions, as shown in Table 2. In previous studies, the web space area (the area between the fingers) was consistently neglected in evaluation of hand anthropometry. Fig 3 shows the measurements of web space angle and the slant of web space from the scanned hand images.

**Table 2:** Hand measurements for glove design.

Category	No.	Dimension	Landmarks
**Circumference**	**C1**	IP joint of D1	25-26-25
	**C2**	Distal IP joint of D2	7-8-7
	**C3**	Distal IP joint of D3	5-6-5
	**C4**	Distal IP joint of D4	3-4-3
	**C5**	Distal IP joint of D5	1-2-1
	**C6**	Proximal IP joint of D2	15-16-15
	**C7**	Proximal IP joint of D3	13-14-13
	**C8**	Proximal IP joint of D4	11-12-11
	**C9**	Proximal IP joint of D5	9-10-9
	**C10**	Finger root of D1	27-28-27
	**C11**	Finger root of D2	23-24-23
	**C12**	Finger root of D3	21-22-21
	**C13**	Finger root of D4	19-20-19
	**C14**	Finger root of D5	17-18-17
	**C15**	Circumference between thumb and palm	34-35-36-34
	**C16**	Hand circumference	32-33-32
	**C17**	Wrist circumference	29-30-31-29
**Length**—**palm**	**L1**	Finger length of D1	Tip of D1-27
	**L2**	Finger length of D2	Tip of D2-23
	**L3**	Finger length of D3	Tip of D3-21
	**L4**	Finger length of D4	Tip of D4-19
	**L5**	Finger length of D5	Tip of D5-17
	**L6**	Length from tip of D1 to wrist-crease	Tip of D1-31
	**L7**	Length from tip of D2 to wrist-crease	Tip of D2-31
	**L8**	Length from tip of D3 to wrist-crease	Tip of D3-31
	**L9**	Length from tip of D4 to wrist-crease	Tip of D4–31
	**L10**	Length from tip of D5 to wrist-crease	Tip of D5–31
	**L11**	Palm length	21–31
	**L12**	Hand breadth	32–33
**Length**—**dorsal**	**L13**	Finger length of D1	Tip of D1-28
	**L14**	Finger length of D2	Tip of D2-24
	**L15**	Finger length of D3	Tip of D3-22
	**L16**	Finger length of D4	Tip of D4-20
	**L17**	Finger length of D5	Tip of D5-18
	**L18**	Length from tip of D1 to wrist-crease	Tip of D1-43
	**L19**	Length from tip of D2 to wrist-crease	Tip of D2-43
	**L20**	Length from tip of D3 to wrist-crease	Tip of D3-43
	**L21**	Length from tip of D4 to wrist-crease	Tip of D4-43
	**L22**	Length from tip of D5 to wrist-crease	Tip of D5-43
	**L23**	Finger root of D2 to MCP joint	24–44
	**L24**	Finger root of D3 to MCP joint	22–45
	**L25**	Finger root of D4 to MCP joint	20–46
	**L26**	Finger root of D5 to MCP joint	18–47
**Length**—**web space**	**L27**	Length between D1 and D2	35–36
	**L28**	Length between D2 and D3	37–38
	**L29**	Length between D3 and D4	39–40
	**L30**	Length between D4 and D5	41–42
**Angle**	**A1**	Web space angle between D1 and D2	26-35-16
	**A2**	Web space angle between D2 and D3	16-37-14
	**A3**	Web space angle between D3 and D4	14-39-12
	**A4**	Web space angle between D4 and D5	12-41-10
	**A5**	Slant of web space between D1 and D2	35-36-31
	**A6**	Slant of web space between D2 and D3	37-38-31
	**A7**	Slant of web space between D3 and D4	39-40-31
	**A8**	Slant of web space between D4 and D5	41-42-31
**Surface area**	**S1**	Finger root to MCP joint	Across 44–47
	**S2**	MCP joint to wrist line	33-32-30-29-28-33

^a^ D: digit; IP: interphalangeal; and MCP: metacarpophalangeal.

#### Dynamic hand postures

**Fig 4:**
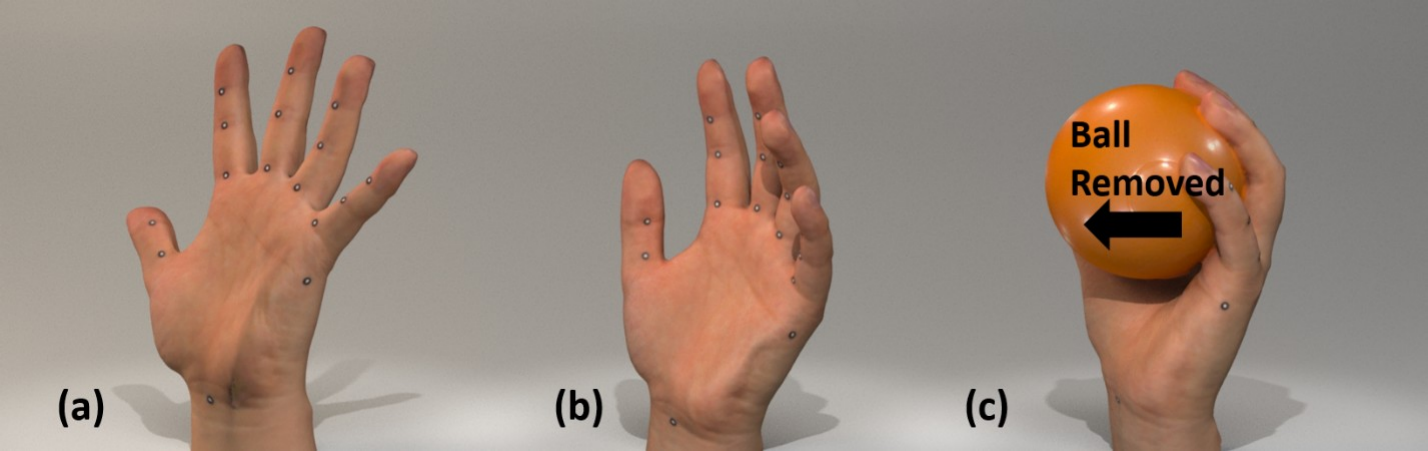
Hand postures. (a) hand with splayed fingers, (b) hand in relaxed state, and (c) ball grip posture.

The left and right hands of each participant were scanned in the splayed, relaxed and gripping hand postures as shown in Fig 4. For the splayed hand posture, the subjects were asked to spread their fingers apart to mimic the standard posture when customizing a glove pattern. For the relaxed hand posture, the subjects were asked to relax their hands as they usually do. For the ball grip posture, the subjects were required to hold a plastic ball with a diameter of 78mm to imitate holding tools, and the ball was removed during scanning for comprehensive measurement. These three postures were selected based on previous references [9, 20].

### Data analysis

The data were analyzed by using Statistical Package for the Social Sciences (SPSS Inc, version 22, IBM, Armonk, NY). All the hand measurements followed normal distribution. An independent samples t-test was conducted to examine the effects of left / right hand on hand measurements. A repeated-measures analysis of variance (rANOVA) was conducted to observe the effect of the independent variables, hand postures (3 levels) taking as within-subject variable and gender taking as between-subject variable, on the dependent variable, 57 hand dimension measurements. The Levene’s tests of equality of variances showed that the variances on all the hand measurements across dependent variables were homogeneous. Data that violated the assumption of sphericity was corrected by using the Huynh-Feldt correction. Sidak pairwise comparisons were performed to compare the mean difference between (1) ball grip and splayed, (2) relaxed and splayed, and (3) relaxed and ball grip postures. Spearman’s correlations among gender, height, weight, body mass index (BMI) and the hand dimension measurements were performed. The significance of the statistical analysis was set at a level of 0.05.

Differences in the skin deformation during the two dynamic postures in comparison to the gripping hand posture were also calculated by using the skin strain ratio, which was determined with the following equation:

λ=[(b−a)/a]×100 (1)

In the equation, λ (%) is the skin strain ratio, *b* (mm) is the mean value of the measurement under a relaxed or splayed posture, and *a* (mm) represents the mean value of each measurement in the gripping posture.

## Results

### Hand measurements

S1 to S3 Tables showed the mean values of 57 dimensions in different postures. The splayed posture is conducive to the length of the palm side, but not conducive to the length of the dorsal side and other measurements in relaxed and gripping postures.

### Differences in hand dimensions among the three postures

The results of independent sample t-test showed that there was no significant difference between the left and right hands in all the measurements. Therefore, the measurements taken from both left and right hands were involved in the rANOVA. The total number of samples became 60 in which 40 hands were from female participants and 20 hands were from male participants. The results of the rANOVA showed that 42 of the 57 hand dimension measurements have significant differences (p < 0.05) among the three types of hand postures, including 6 circumferential dimensions, 26 length dimensions, 6 angle measurements and the 2 surface area measurements. Table 3 summarizes the results of the repeated-measures ANOVA. Pairwise comparisons showed that 36 of the dimensions have significant differences between ball grip and splayed postures, 34 were significantly difference between relaxed and splayed postures, and 32 have significant differences between relaxed and ball grip postures. Moreover, there were significant differences between the two genders in circumference, length, and surface area dimensions, but no significant differences in all the angular dimensions.

**Table 3:** Summary of rANOVA results for effect of three postures on each hand measurement (n = 60).

No.	p-value	ƞ2	No.	p-value	ƞ2
**C1**	0.65	0.01	L13	0.00	0.49
**C2**	0.69	0.01	L14	0.00	0.74
**C3**	0.29	0.02	L15	0.00	0.62
**C4**	0.26	0.02	L16	0.00	0.61
**C5**	0.01	0.08	L17	0.00	0.66
**C6**	0.53	0.01	L18	0.00	0.75
**C7**	0.37	0.02	L19	0.00	0.44
**C8**	0.61	0.01	L20	0.00	0.39
**C9**	0.02	0.07	L21	0.00	0.48
**C10**	0.58	0.01	L22	0.00	0.59
**C11**	0.17	0.03	L23	0.00	0.13
**C12**	0.20	0.03	L24	0.00	0.11
**C13**	0.02	0.06	L25	0.03	0.06
**C14**	0.04	0.06	L26	0.00	0.53
**C15**	0.28	0.02	L27	0.05	0.05
**C16**	0.00	0.23	L28	0.90	0.00
**C17**	0.00	0.26	L29	0.00	0.13
**L1**	0.00	0.15	L30	0.55	0.01
**L2**	0.00	0.28	A1	0.00	0.27
**L3**	0.00	0.60	A2	0.30	0.02
**L4**	0.00	0.74	A3	0.00	0.19
**L5**	0.00	0.81	A4	0.00	0.22
**L6**	0.00	0.75	A5	0.02	0.07
**L7**	0.00	0.68	A6	0.00	0.73
**L8**	0.00	0.74	A7	0.00	0.46
**L9**	0.00	0.61	A8	0.00	0.65
**L10**	0.00	0.67	S1	0.00	0.33
**L11**	0.00	0.82	S2	0.00	0.29
**L12**	0.00	0.81			

^a^ Significant value (p < 0.05) are shaded grey.

### Relationship among gender, height, weight, BMI and hand dimension measurements

The results of the Spearman’s correlations showed that more than 88% of the circumference, length, and surface area dimensions were significantly and positively correlated to gender, height, weight, and BMI, respectively. However, less than half of the angle measurements were significantly positively correlated with these factors.

### Skin deformation between the three postures

**Fig 5:**
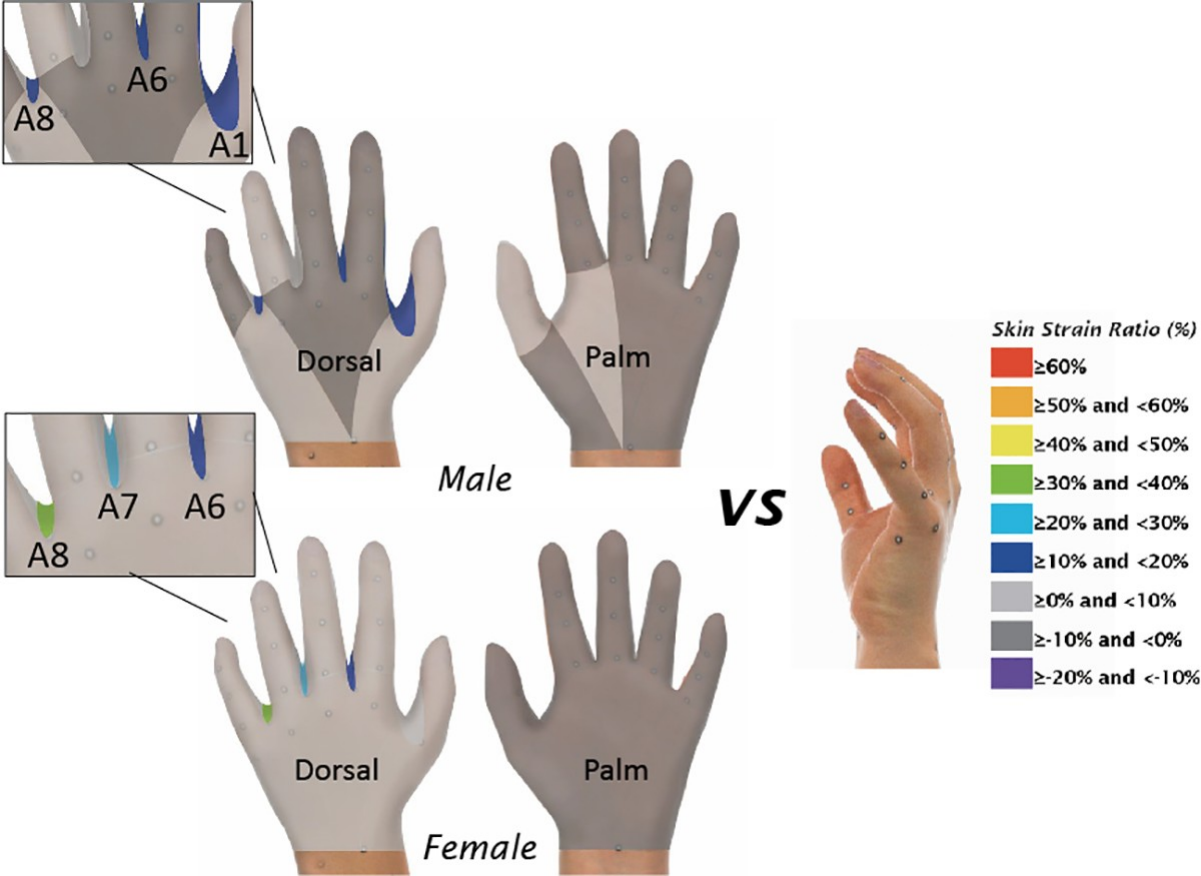
Skin strain ratio from gripping to relaxed posture.

**Fig 6:**
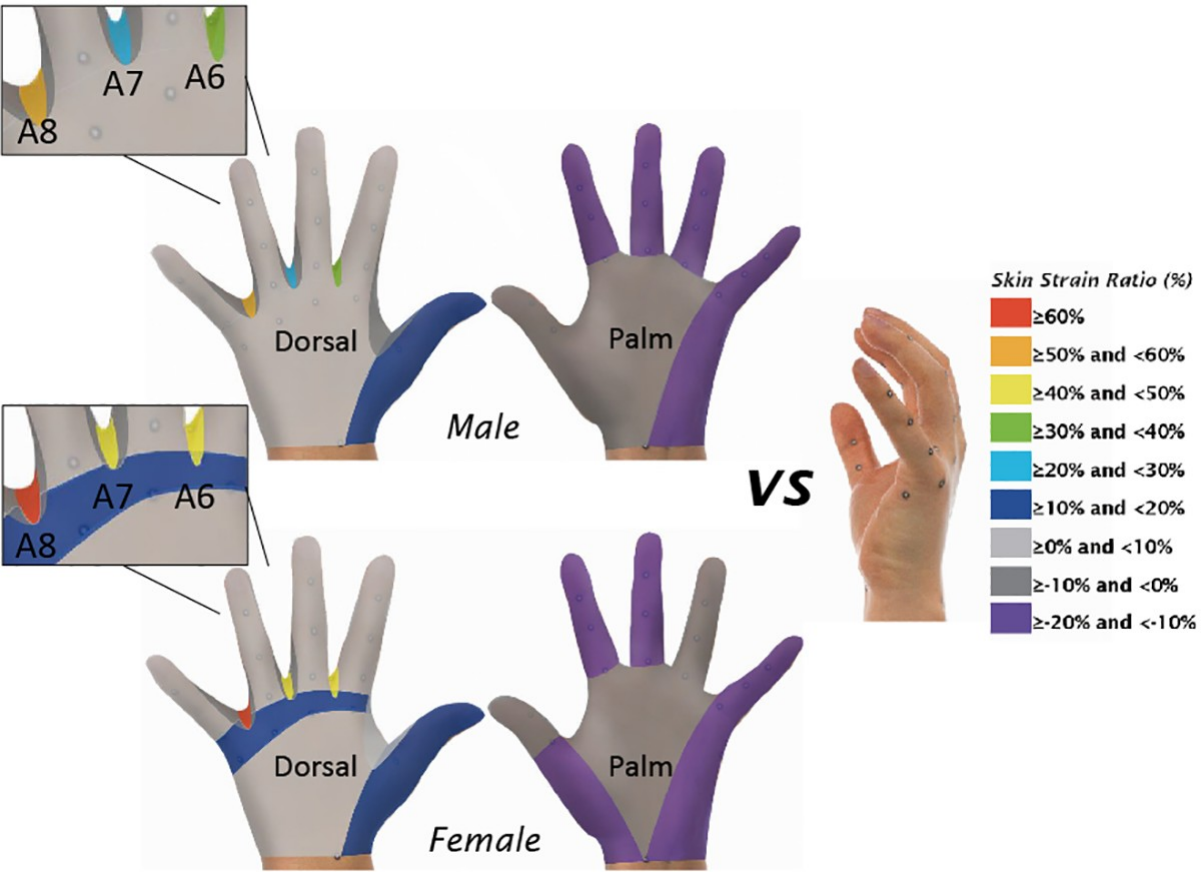
Skin strain ratio from gripping to splayed posture.

The skin strain ratios were also calculated and compared to assess the skin deformation among the different postures. Table 4, Figs 5 and 6 show the skin strain ratios of the 42 hand measurements obtained from the female and male subjects respectively. Based on the equation, a negative skin strain ratio means that the dimension has a higher value in the ball grip posture than the relaxed or splayed postures and vice versa. The skin strain ratios between the ball grip and the relaxed postures, and between the ball grip and the splayed postures ranged from -6.6% to 30.8% and from -12.2% to 65.5% respectively. As compared to the ball grip posture, the level of skin deformation obtained from the splayed hand posture is consistently higher than the relaxed posture, especially in the slants of web space (A6, A7 and A8) and the length dimensions. Results also indicated that 85% of the female measurements and 87.5% of the male measurements have greater skin deformation when changed from gripping to the splayed posture when compared to the relaxed posture. The skin strain ratio of most palm-length dimensions (L1-L12) is negative, while the skin strain ratio of most dorsal-length (L13-L26) is positive.

**Table 4:** Skin strain ratio (%) of hand measurement.

No.	Dimension	Female skin strain ratio (%) (N = 40)		Male skin strain ratio (%) (N = 20)	
		Ball grip vs Relaxed	Ball grip vs Splayed	Ball grip vs Relaxed	Ball grip vs Splayed
**C5**	Distal IP joint of D5	2.22	1.06	1.23	3.32
**C9**	Proximal IP joint of D5	1.60	0.80	0.38	2.23
**C13**	Finger root of D4	-1.29	-1.85	0.73	-1.29
**C14**	Finger root of D5	0.02	-2.47	0.55	-0.21
**C16**	Hand circumference	1.91	1.62	1.10	0.96
**C17**	Wrist circumference	-0.26	-1.58	0.02	-1.30
**L1**	Finger length of D1	-0.32	-7.58	0.24	-9.80
**L2**	Finger length of D2	-3.19	‐10.17	-3.28	‐11.47
**L3**	Finger length of D3	-3.89	‐10.90	-4.16	‐12.18
**L4**	Finger length of D4	-3.24	-9.82	-2.38	‐10.16
**L5**	Finger length of D5	-4.12	‐11.58	-3.61	‐11.13
**L6**	Length from tip of D1 to wrist-crease	-4.37	‐10.62	-3.07	-9.88
**L7**	Length from tip of D2 to wrist-crease	-0.29	-4.54	0.00	-4.73
**L8**	Length from tip of D3 to wrist-crease	-2.09	-6.23	-1.59	-5.54
**L9**	Length from tip of D4 to wrist-crease	-3.01	-7.28	-1.83	-6.81
**L10**	Length from tip of D5 to wrist-crease	-6.61	‐10.94	-5.32	‐10.07
**L11**	Palm length	-1.11	-2.60	0.35	-0.91
**L12**	Hand breadth	4.57	6.50	3.37	4.60
**L13**	Finger length of D1	1.04	7.79	0.40	10.67
**L14**	Finger length of D2	1.03	8.86	0.85	9.01
**L15**	Finger length of D3	0.43	6.98	-0.59	7.83
**L16**	Finger length of D4	1.76	8.72	0.68	8.06
**L17**	Finger length of D5	0.37	8.02	-1.19	9.71
**L18**	Length from tip of D1 to wrist-crease	5.01	10.86	4.67	10.81
**L19**	Length from tip of D2 to wrist-crease	0.45	3.45	-0.03	2.99
**L20**	Length from tip of D3 to wrist-crease	0.09	2.75	-1.56	2.47
**L21**	Length from tip of D4 to wrist-crease	0.92	4.03	-0.81	3.13
**L22**	Length from tip of D5 to wrist-crease	2.67	6.70	2.29	6.61
**L23**	Finger root of D2 to MCP joint	4.39	7.95	2.72	4.23
**L24**	Finger root of D3 to MCP joint	-0.20	3.29	-1.38	4.42
**L25**	Finger root of D4 to MCP joint	-2.04	2.67	2.46	3.82
**L26**	Finger root of D5 to MCP joint	10.93	22.52	9.29	19.43
**L29**	Length between D3 and D4	-1.09	-6.74	2.64	-7.11
**A1**	Web space angle between D1 and D2	8.83	0.67	10.01	-4.69
**A3**	Web space angle between D3 and D4	7.34	-2.22	2.02	-9.62
**A4**	Web space angle between D4 and D5	9.13	-1.71	9.17	-3.29
**A5**	Slant of web space between D1 and D2	1.73	0.48	8.18	-4.43
**A6**	Slant of web space between D2 and D3	19.75	41.19	14.22	38.11
**A7**	Slant of web space between D3 and D4	23.24	40.66	7.71	29.13
**A8**	Slant of web space between D4 and D5	30.85	65.49	19.48	51.38
**S1**	Finger root to MCP joint	8.80	10.96	3.02	7.64
**S2**	MCP joint to wrist line	6.15	7.14	4.28	3.33

^a^ Skin strain ratio of ≥ ± 10% are shaded grey.

## Discussion

This study adopted 3D scanning method to investigate the influence of posture variation on hand dimensions. The results indicate that hand posture has a major impact on hand measurements, hence the glove pattern design. About two-thirds of the hand dimensions change significantly during hand movements, including hand lengths, web space angles and surface areas. Consequently, the current glove pattern design based on hand dimensions obtained from the standard splayed posture fails to accommodate the hand in motion.

### Skin deformation between the three postures

Amongst the four categories of hand measurements, skin deformation among the circumferential dimensions is the lowest (-2.5% to 3.3%), whilst the greatest amount of skin deformation is found among the angular dimensions (-9.6% to 65.5%). Since conventional glove pattern design merely refers to the length and circumference of the hand, most of the earlier studies in the literature have only focused on the changes in the hand circumference and length during hand movement [9, 13, 20]. The influence of hand posture on the changes in the angular dimensions has not been fully reported in the literature. Gloves based on the current dimensions of the splayed posture regardless of the web space angle measurements and the corresponding changes in posture therefore inevitably limit hand movement.

The length dimensions also considerably changed with postures, with skin strain ratio ranged from -12.2% to 22.5%. The hand dorsal-length measurements mostly increased, whilst the palm-length measurements reduced due to hand contraction. The assumption of identical length dimensions of the dorsal and the palm side in conventional glove design therefore inherently results in fit and comfort problems. In this study, the influence of the hand postures on finger dimensions is apparent. Previous studies focused primarily on the dimensions of the thumb since humans use the thumb more often [23], therefore failed to fully address the glove fit problem. Discomfort in the palm area and fingers has been reported when wearing gloves for the plier task, and the discomfort increases with time [24]. The dimension changes of the fingers should be considered in engineering design of gloves to boost the fit and comfort.

Apart from the length and angular dimensions, the surface area also has skin deformation with both the relaxed and splayed postures (3.0% to 11.0%). The increased dimensions and surface area on the dorsal side in gripping may lead to increased stress between the hand and the glove so that the wearer may experience stretch-difficulties, discomfort or even obstruct blood circulation. The surface area between the finger root to the MCP joint (S1) changes more among the postures as opposed to the surface area between the MCP joint to wrist line (S2). This indicates that skin deformation is significantly greater in the metacarpal region, and the curvature of the bones in the metacarpal region is more pronounced. Tolerance of the surface area between the finger root to the MCP joint and between the MCP joint to the wrist line should be determined with reference to the skin strain ratios to advance glove pattern designs for optimum fit.

In this study, the overall skin strain ratios obtained from the relaxed and the splayed postures vary from -12.2% to 65.5% as compared to the ball grip posture. Hand postures cause high levels of skin deformation and substantial changes in hand dimensions. Conventional glove pattern design based on hand dimensions with a splayed posture therefore inevitably restricts the movement of gloved hands, especially when gripping an object.

### Hand dimensions and gender, height, weight, BMI

Spearman’s correlation results show that gender, height, weight, and BMI are significantly correlated with most of the circumference, length, and surface area measurements, respectively. The height, weight and BMI of the male subjects are always higher than those of the female subjects. These factors may lead to larger mean values of the hand circumference, length, and surface area. Interestingly, gender, height, weight, and BMI have no correlations with more than half of the angle measurements, including the angle and slant of the web spaces. The results of rANOVA showed that there were no significant differences between the two genders in all the angle dimensions. This may show that the geometric changes of the web space are not affected by gender or size of the hand.

In addition, there was no significant different between the left and right hands and all the measurements. This can be inferred that the hand measurements are not affected by the dominant hand. In previous research on gloves or hand measurements, the dominance of hand was always taken into consideration [9, 12, 20, 25]. This study shows that fabricating gloves with the same dimensions for the left and right hands does not cause significant fitting problems. This helps to reduce considerations for glove customization. This may further address the myth that there is a substantial difference between the dimensions of the left and right hands.

### Application on glove pattern design

To allow free movement of hands, ease allowance (ranging from 29% to 65%) should be added to the areas of the slant of the web spaces between D2 to D5 in glove patterns. Considering the skin strain ratio in finger lengths, the finger length of the palm side should be reduced by around 10% to better fit the palm and facilitate a gripping posture. On the other hand, the finger length of the dorsal side should be increased by an average of 9% or using a fabric with enough extensibility to accommodate the increase of hand dimensions, allowing flexible hand movement with minimal stress on the skin.

### Limitation and recommendation

The sample size of this study is relatively small and most of them are in the same glove size of M based on their hand circumference, therefore might have limited the generalizability of the results. Future studies involving a larger number of subjects with various glove size should follow. Nonetheless, the study provides preliminary evidence that supports the significant changes in hand angular dimensions and skin surface morphology, especially for the slant of the web space relevant to hand postures, thereby providing a basis for future studies to advance the design and construction of functional gloves with optimal fit, performance and comfort.

## Conclusions

The fit of gloves is important because it affects the safety, performance, and comfort of the wearer. Gloves currently constructed based on a splayed posture cannot provide a good fit. Consideration should be given to hand measurements in dynamic postures. In response, this study incorporates 3D scanning technology to analyze skin deformation with two dynamic postures as well as the static splayed posture. Detailed measurements can help to produce a well-fitting glove. This study has demonstrated the usefulness of 3D scanning of the hand in different hand postures and successfully obtained various hand measurements which are difficult to directly measure such as the angle of the web space and the hand surface areas. This creates the possibility of computer automatic generation of a glove pattern with improved fit based on 3D hand images.

Amongst the three different types of hand postures, two-thirds of the hand dimensions significantly change during hand movement, including most of the hand lengths, angles of the web space and surface areas of the metacarpophalangeal joint. Conventional glove pattern designs which are based on hand dimensions with a splayed posture therefore limit the movement of gloved hands, particularly with a gripping posture. It is recommended to increase the inclination of the web space between D2 and D5, reduce the length of the fingers on the palm side, and increase the length of the fingers on the dorsal side. The results obtained from this study not only address the intricacies of gloves by incorporating precise hand measurements and different postures, but also provide insights into glove designs and fit that enable better hand protection and comfort. The skin strain ratios can be used for comparison with the nominal plots of the stress-stretch ratio of different types of fabrics for gloves to examine the effect of the fabric properties, and further develop gloves with materials that cater to different occupations and professions. Skin strain ratio with consideration of hand dimension changes in posture variation should be referenced to advance glove pattern designs for optimum fit.

## Supporting information

S1 Table Mean value and standard deviation of the hand measurement in female group (n = 40). (DOCX)

S2 Table Mean value and standard deviation of the hand measurement in male group (n = 20). (DOCX)

S3 Table Overall mean value and standard deviation of the hand measurement (n = 60). (DOCX)

S1 File (DOCX)

## References

[1] Akbar-Khanzadeh F, Bisesi MS, Rivas RD. Comfort of personal protective equipment. Applied Ergonomics. 1995;26(3):195–8. 10.1016/0003-6870(95)00017-7 15677018

[2] Barker RL, Ross KA, Andrews J, Deaton AS. Comparative studies on standard and new test methods for evaluating the effects of structural firefighting gloves on hand dexterity. Textile Research Journal. 2017;87(3):270–84. 10.1177/0040517516629143

[3] Dunbar B, Chapates P, editors. Comparison of 3D Photogrammetric and Laser Hand Scans to Manual Measurement Methods for EVA Glove Fabrication. 2019 IEEE Aerospace Conference; 2019: IEEE.

[4] Hsiao H, Whitestone J, Kau T-Y, Hildreth B. Firefighter hand anthropometry and structural glove sizing: a new perspective. Human Factors. 2015;57(8):1359–77. 10.1177/0018720815594933 26169309 PMC4681492

[5] Opperman RA, Waldie J, Natapoff A, Newman DJ, Jones JA. Probability of spacesuit-induced fingernail trauma is associated with hand circumference. Aviation Space and Environmental Medicine. 2010;81(10):907–13. 10.3357/asem.2810.2010 20922881

[6] Reid CR, McFarland SM. Feasibility assessment of an EVA glove sensing platform to evaluate potential hand injury risk factors. International Conference on Environmental Systems. 2015.

[7] Yu A, Yick K, Ng S, Yip J, Zhang X. Modification of finger web gusset for improving fit and comfort of pressure therapy gloves. Textile bioengineering and informatics symposium proceedings. 2014.

[8] Mohammadian M, Choobineh A, Haghdoost AA, Hashemi Nejad N. Investigation of grip and pinch strengths in Iranian adults and their correlated anthropometric and demographic factors. Work. 2016;53(2):429–37. 10.3233/WOR-152180 26519018

[9] Nasir SH, Troynikov O. Influence of hand movement on skin deformation: A therapeutic glove design perspective. Applied Ergonomics. 2017;60:154–62. 10.1016/j.apergo.2016.11.006 28166874

[10] Choi S, Ashdown SP. 3D body scan analysis of dimensional change in lower body measurements for active body positions. Textile research journal. 2010;81(1):81–93. 10.1177/0040517510377822

[11] Lee J, Ashdoon SP. Upper body surface change analysis using 3-D body scanner. Journal of the Korean Society of Clothing and Textiles. 2005;29(12):1595–607.

[12] Griffin L, Kim N, Carufel R, Sokolowski S, Lee H, Seifert E, editors. Dimensions of the dynamic hand: implications for glove design, fit, and sizing. International Conference on Applied Human Factors and Ergonomics; 2018: Springer.

[13] Kwon O, Jung K, You H, Kim H-E. Determination of key dimensions for a glove sizing system by analyzing the relationships between hand dimensions. Applied Ergonomics. 2009;40(4):762–6. 10.1016/j.apergo.2008.07.003 18799156

[14] Holt GR. Declaration of Helsinki—The World’s Document of Conscience and Responsibility. Southern medical journal. 2014;107(7):407–. 10.14423/SMJ.0000000000000131 25010578

[15] Robinette KM, Daanen H, Paquet E, editors. The CAESAR project: a 3-D surface anthropometry survey. Second International Conference on 3-D Digital Imaging and Modeling; 1999: IEEE.

[16] Seminati E, Canepa Talamas D, Young M, Twiste M, Dhokia V, Bilzon JL. Validity and reliability of a novel 3D scanner for assessment of the shape and volume of amputees’ residual limb models. PLoS One. 2017;12(9):e0184498. 10.1371/journal.pone.0184498 28886154 PMC5590959

[17] Stewart A, Ledingham R, Williams H. Variability in body size and shape of UK offshore workers: A cluster analysis approach. Applied Ergonomics. 2017;58:265–72. 10.1016/j.apergo.2016.07.001 27633221

[18] Wan FK, Yick K-L, Winnie W. Validation of a 3D foot scanning system for evaluation of forefoot shape with elevated heels. Measurement. 2017;99:134–44.

[19] Yu A, Yick KL, Ng SP, Yip J, Chan YF. Numerical simulation of pressure therapy glove by using Finite Element Method. Burns. 2016;42(1):141–51. 10.1016/j.burns.2015.09.013 26520450

[20] Nasir SH, Troynikov O, Watson C. Skin deformation behavior during hand movements and their impact on functional sports glove design. Procedia Engineering. 2015;112:92–7.

[21] Modabber A, Peters F, Kniha K, Goloborodko E, Ghassemi A, Lethaus B, et al. Evaluation of the accuracy of a mobile and a stationary system for three-dimensional facial scanning. Journal of Cranio-Maxillofacial Surgery. 2016;44(10):1719–24. 10.1016/j.jcms.2016.08.008 27614543

[22] Verhulst A, Hol M, Vreeken R, Becking A, Ulrich D, Maal T. Three-dimensional imaging of the face: a comparison between three different imaging modalities. Aesthetic Surgery Journal. 2018;38(6):579–85. 10.1093/asj/sjx227 29360971

[23] Yamada Y, Morizono T, Sato K, Shibuya H, Shimohira T, Umetani Y, et al. Proposal of a skilmate hand and its component technologies for extravehicular activity gloves. Advanced Robotics. 2004;18(3):269–84.

[24] Dianat I, Haslegrave CM, Stedmon AW. Using pliers in assembly work: Short and long task duration effects of gloves on hand performance capabilities and subjective assessments of discomfort and ease of tool manipulation. Applied Ergonomics. 2012;43(2):413–23. 10.1016/j.apergo.2011.06.016 21777904

[25] Dargan D, Mandal A, Shokrollahi K. Hand burns surface area: A rule of thumb. Burns. 2018;44(5):1346–51. 10.1016/j.burns.2018.02.011 29534883

