# Supplementary material for: Impact of postural variation on hand measurements: Three-dimensional anatomical analysis
Source: PLoS One. 2021 Apr 23;16(4):e0250428. doi: 10.1371/journal.pone.0250428 (PMC8064611; doi:10.1371/journal.pone.0250428)
Supplement: S1 Table — (DOCX) [file pone.0250428.s001.docx]

**S1 Table. Mean value and standard deviation of the hand measurement in female group (n=40)**

|  |  | **Relaxed** | | **Ball grip** | | **Splayed** | |
| --- | --- | --- | --- | --- | --- | --- | --- |
|  |  | **Mean** | **S.D.** | **Mean** | **S.D.** | **Mean** | **S.D.** |
| **Circumference (mm)** | **C1** | 57.58 | 3.85 | 57.66 | 3.37 | 57.11 | 3.13 |
|  | **C2** | 45.35 | 2.74 | 45.57 | 2.44 | 45.15 | 2.31 |
|  | **C3** | 45.68 | 2.84 | 45.75 | 2.20 | 45.45 | 2.39 |
|  | **C4** | 42.76 | 2.55 | 42.82 | 2.18 | 42.73 | 2.25 |
|  | **C5** | 39.05 | 2.82 | 39.91 | 2.39 | 39.49 | 2.04 |
|  | **C6** | 54.55 | 2.71 | 54.65 | 2.53 | 54.40 | 2.45 |
|  | **C7** | 56.19 | 2.66 | 56.33 | 2.61 | 55.63 | 2.73 |
|  | **C8** | 53.16 | 2.55 | 53.17 | 2.56 | 52.86 | 2.52 |
|  | **C9** | 46.51 | 3.16 | 47.25 | 2.85 | 46.88 | 2.34 |
|  | **C10** | 66.63 | 6.46 | 66.17 | 6.11 | 66.64 | 6.09 |
|  | **C11** | 61.57 | 4.06 | 61.24 | 4.11 | 61.39 | 3.76 |
|  | **C12** | 59.76 | 3.74 | 60.11 | 4.15 | 59.85 | 4.03 |
|  | **C13** | 58.36 | 3.77 | 57.61 | 4.12 | 58.69 | 3.88 |
|  | **C14** | 53.73 | 4.27 | 53.74 | 4.75 | 55.11 | 4.18 |
|  | **C15** | 128.10 | 8.58 | 127.50 | 8.65 | 128.64 | 7.54 |
|  | **C16** | 182.99 | 8.06 | 186.49 | 8.34 | 183.52 | 8.50 |
|  | **C17** | 151.63 | 5.26 | 151.24 | 5.04 | 153.66 | 5.35 |
| **Length - palm (mm)** | **L1** | 50.10 | 4.11 | 49.94 | 3.53 | 54.03 | 3.55 |
|  | **L2** | 64.93 | 4.01 | 62.86 | 2.99 | 69.98 | 4.04 |
|  | **L3** | 72.65 | 4.43 | 69.83 | 3.35 | 78.37 | 3.63 |
|  | **L4** | 67.82 | 4.48 | 65.62 | 3.77 | 72.77 | 4.18 |
|  | **L5** | 54.44 | 4.47 | 52.19 | 3.69 | 59.02 | 4.25 |
|  | **L6** | 123.27 | 7.29 | 117.88 | 5.47 | 131.88 | 5.98 |
|  | **L7** | 166.49 | 7.64 | 166.01 | 6.09 | 173.92 | 6.53 |
|  | **L8** | 174.75 | 7.56 | 171.10 | 6.91 | 182.46 | 6.67 |
|  | **L9** | 163.76 | 8.62 | 158.83 | 6.60 | 171.31 | 8.01 |
|  | **L10** | 140.05 | 8.20 | 130.80 | 7.82 | 146.87 | 7.45 |
|  | **L11** | 102.25 | 5.07 | 101.12 | 4.99 | 103.82 | 5.27 |
|  | **L12** | 100.01 | 6.61 | 104.58 | 6.05 | 98.19 | 5.93 |
| **Length - dorsal (mm)** | **L13** | 54.23 | 5.93 | 54.79 | 6.42 | 50.84 | 5.30 |
|  | **L14** | 77.14 | 4.78 | 77.94 | 4.36 | 71.60 | 3.84 |
|  | **L15** | 86.26 | 5.54 | 86.63 | 5.57 | 80.98 | 5.33 |
|  | **L16** | 79.66 | 5.04 | 81.07 | 6.12 | 74.57 | 5.35 |
|  | **L17** | 62.22 | 5.41 | 62.45 | 5.77 | 57.82 | 4.60 |
|  | **L18** | 132.64 | 7.13 | 139.29 | 7.57 | 125.65 | 6.79 |
|  | **L19** | 166.09 | 8.30 | 166.84 | 7.55 | 161.28 | 7.84 |
|  | **L20** | 173.04 | 8.72 | 173.19 | 8.96 | 168.55 | 8.05 |
|  | **L21** | 164.24 | 9.05 | 165.76 | 9.90 | 159.34 | 8.11 |
|  | **L22** | 145.14 | 7.85 | 149.02 | 8.93 | 139.66 | 7.65 |
|  | **L23** | 15.91 | 4.77 | 16.61 | 5.17 | 15.38 | 4.63 |
|  | **L24** | 16.60 | 5.05 | 16.57 | 5.32 | 16.04 | 4.97 |
|  | **L25** | 16.88 | 6.07 | 17.23 | 5.94 | 16.78 | 5.98 |
|  | **L26** | 16.36 | 5.17 | 18.15 | 6.09 | 14.81 | 4.74 |
| **Length - web space (mm)** | **L27** | 13.09 | 2.16 | 13.10 | 2.48 | 12.63 | 2.09 |
|  | **L28** | 12.01 | 2.48 | 12.36 | 2.32 | 12.42 | 1.77 |
|  | **L29** | 11.61 | 1.98 | 11.48 | 1.98 | 12.31 | 2.61 |
|  | **L30** | 10.95 | 1.94 | 11.38 | 1.65 | 11.19 | 1.64 |
| **Angle (degree)** | **A1** | 80.53 | 12.06 | 87.64 | 7.77 | 87.06 | 11.53 |
|  | **A2** | 60.71 | 10.92 | 60.63 | 8.61 | 61.03 | 7.77 |
|  | **A3** | 49.44 | 5.94 | 53.06 | 8.24 | 54.27 | 6.11 |
|  | **A4** | 62.49 | 9.75 | 68.20 | 10.92 | 69.39 | 8.60 |
|  | **A5** | 61.87 | 8.41 | 60.80 | 8.03 | 60.51 | 7.29 |
|  | **A6** | 59.13 | 9.91 | 70.81 | 8.94 | 50.15 | 8.06 |
|  | **A7** | 48.62 | 11.60 | 59.92 | 11.85 | 42.60 | 8.67 |
|  | **A8** | 57.22 | 10.48 | 74.87 | 15.74 | 45.24 | 10.27 |
| **Surface area (mm^2^)** | **S1** | 1877.21 | 548.18 | 2042.45 | 602.52 | 1840.69 | 536.69 |
|  | **S2** | 6502.29 | 549.15 | 6902.05 | 531.95 | 6442.39 | 511.26 |

^a^ Higher value are shaded grey
