# Supplementary material for: Impact of postural variation on hand measurements: Three-dimensional anatomical analysis
Source: PLoS One. 2021 Apr 23;16(4):e0250428. doi: 10.1371/journal.pone.0250428 (PMC8064611; doi:10.1371/journal.pone.0250428)
Supplement: S2 Table — (DOCX) [file pone.0250428.s002.docx]

**S2 Table. Mean value and standard deviation of the hand measurement in male group (n=20)**

|  |  | | **Relaxed** | | | **Ball grip** | | | **Splayed** | | |
| --- | --- | --- | --- | --- | --- | --- | --- | --- | --- | --- | --- |
|  |  | | **Mean** | **S.D.** | | **Mean** | **S.D.** | | **Mean** | **S.D.** | |
| **Circumference (mm)** | | **C1** | 65.34 | | 3.41 | 65.36 | | 3.40 | 65.20 | | 3.58 |
|  |  | **C2** | 51.43 | | 2.51 | 51.72 | | 2.40 | 51.96 | | 2.87 |
|  |  | **C3** | 51.87 | | 2.89 | 52.71 | | 2.81 | 52.87 | | 3.22 |
|  |  | **C4** | 48.48 | | 3.16 | 48.70 | | 2.94 | 49.47 | | 3.57 |
|  |  | **C5** | 45.39 | | 2.71 | 45.95 | | 2.72 | 44.48 | | 2.94 |
|  |  | **C6** | 61.17 | | 2.76 | 60.68 | | 2.07 | 61.41 | | 2.68 |
|  |  | **C7** | 62.18 | | 2.98 | 62.78 | | 2.64 | 63.11 | | 3.53 |
|  |  | **C8** | 58.90 | | 2.67 | 59.35 | | 2.54 | 59.70 | | 2.89 |
|  |  | **C9** | 53.32 | | 3.00 | 53.52 | | 2.87 | 52.35 | | 2.47 |
|  |  | **C10** | 72.78 | | 5.10 | 72.59 | | 4.68 | 73.30 | | 4.73 |
|  |  | **C11** | 67.85 | | 3.82 | 67.15 | | 3.13 | 68.02 | | 3.00 |
|  |  | **C12** | 64.55 | | 2.80 | 65.09 | | 3.22 | 65.39 | | 3.58 |
|  |  | **C13** | 61.28 | | 3.76 | 61.73 | | 3.83 | 62.53 | | 3.63 |
|  |  | **C14** | 58.06 | | 3.81 | 58.38 | | 3.86 | 58.50 | | 3.68 |
|  |  | **C15** | 146.35 | | 7.41 | 148.94 | | 11.25 | 148.54 | | 9.82 |
|  |  | **C16** | 206.77 | | 8.58 | 209.04 | | 8.49 | 207.06 | | 8.42 |
|  |  | **C17** | 167.83 | | 5.37 | 167.86 | | 7.17 | 170.07 | | 6.04 |
| **Length - palm (mm)** | | **L1** | 53.98 | | 3.19 | 54.11 | | 4.08 | 59.99 | | 3.48 |
|  |  | **L2** | 70.98 | | 2.52 | 68.65 | | 3.77 | 77.55 | | 3.39 |
|  |  | **L3** | 79.17 | | 2.57 | 75.88 | | 3.03 | 86.40 | | 3.64 |
|  |  | **L4** | 73.40 | | 2.86 | 71.65 | | 2.40 | 79.75 | | 2.46 |
|  |  | **L5** | 60.05 | | 3.72 | 57.88 | | 4.22 | 65.13 | | 3.71 |
|  |  | **L6** | 134.07 | | 7.65 | 129.95 | | 7.46 | 144.20 | | 8.37 |
|  |  | **L7** | 182.93 | | 6.23 | 182.93 | | 7.26 | 192.02 | | 7.88 |
|  |  | **L8** | 191.49 | | 6.38 | 188.44 | | 6.32 | 199.50 | | 9.45 |
|  |  | **L9** | 179.32 | | 6.97 | 176.03 | | 6.36 | 188.88 | | 7.25 |
|  |  | **L10** | 155.29 | | 7.48 | 147.02 | | 8.25 | 163.48 | | 8.75 |
|  |  | **L11** | 112.88 | | 6.15 | 113.28 | | 5.47 | 114.31 | | 6.40 |
|  |  | **L12** | 116.14 | | 5.50 | 120.05 | | 9.56 | 114.77 | | 5.54 |
| **Length - dorsal (mm)** | | **L13** | 59.36 | | 4.64 | 59.60 | | 4.56 | 53.85 | | 4.67 |
|  |  | **L14** | 82.35 | | 5.17 | 83.05 | | 3.84 | 76.19 | | 3.92 |
|  |  | **L15** | 91.72 | | 4.45 | 91.19 | | 4.09 | 84.57 | | 5.41 |
|  |  | **L16** | 84.14 | | 4.33 | 84.71 | | 5.56 | 78.40 | | 5.13 |
|  |  | **L17** | 68.54 | | 6.09 | 67.72 | | 6.17 | 61.73 | | 5.75 |
|  |  | **L18** | 148.36 | | 8.97 | 155.29 | | 6.95 | 140.14 | | 6.90 |
|  |  | **L19** | 183.28 | | 8.93 | 183.24 | | 8.63 | 177.91 | | 9.02 |
|  |  | **L20** | 192.76 | | 8.74 | 189.75 | | 8.20 | 185.17 | | 8.46 |
|  |  | **L21** | 183.24 | | 8.34 | 181.75 | | 8.70 | 176.24 | | 8.43 |
|  |  | **L22** | 163.52 | | 12.08 | 167.26 | | 8.45 | 156.90 | | 7.91 |
|  |  | **L23** | 20.97 | | 2.54 | 21.54 | | 3.01 | 20.67 | | 3.10 |
|  |  | **L24** | 23.26 | | 3.63 | 22.94 | | 3.26 | 21.97 | | 4.18 |
|  |  | **L25** | 23.01 | | 3.46 | 22.45 | | 3.40 | 21.62 | | 3.24 |
|  |  | **L26** | 20.88 | | 4.55 | 22.82 | | 3.59 | 19.11 | | 2.67 |
| **Length - web space (mm)** | | **L27** | 15.17 | | 2.54 | 15.41 | | 2.64 | 14.48 | | 3.18 |
|  |  | **L28** | 14.55 | | 2.26 | 14.19 | | 2.41 | 14.34 | | 2.14 |
|  |  | **L29** | 13.11 | | 2.05 | 13.46 | | 2.93 | 14.49 | | 2.45 |
|  |  | **L30** | 13.29 | | 2.58 | 13.31 | | 2.55 | 13.07 | | 2.66 |
| **Angle (degree)** | | **A1** | 80.16 | | 15.17 | 88.18 | | 7.36 | 92.52 | | 12.45 |
|  |  | **A2** | 56.94 | | 10.30 | 60.11 | | 8.13 | 59.75 | | 6.95 |
|  |  | **A3** | 47.69 | | 9.75 | 48.66 | | 6.55 | 53.84 | | 6.55 |
|  |  | **A4** | 60.52 | | 7.97 | 66.07 | | 10.87 | 68.32 | | 9.60 |
|  |  | **A5** | 66.67 | | 10.04 | 61.22 | | 6.35 | 64.05 | | 7.41 |
|  |  | **A6** | 60.69 | | 8.19 | 69.32 | | 5.32 | 50.19 | | 6.17 |
|  |  | **A7** | 52.67 | | 7.93 | 56.73 | | 9.17 | 43.93 | | 6.02 |
|  |  | **A8** | 61.01 | | 6.71 | 72.89 | | 11.14 | 48.15 | | 7.02 |
| **Surface area (mm^2^)** | | **S1** | 2834.55 | | 445.35 | 2920.13 | | 377.65 | 2712.97 | | 476.82 |
|  |  | **S2** | 8218.63 | | 842.95 | 8570.47 | | 803.31 | 8294.19 | | 739.61 |

^a^ Higher value are shaded grey
