# Supplementary material for: Impact of postural variation on hand measurements: Three-dimensional anatomical analysis
Source: PLoS One. 2021 Apr 23;16(4):e0250428. doi: 10.1371/journal.pone.0250428 (PMC8064611; doi:10.1371/journal.pone.0250428)
Supplement: S3 Table — (DOCX) [file pone.0250428.s003.docx]

**S3 Table. Overall mean value and standard deviation of the hand measurement (n=60)**

|  |  | **Relaxed** | | **Ball grip** | | **Splayed** | |
| --- | --- | --- | --- | --- | --- | --- | --- |
|  |  | **Mean** | **S.D.** | **Mean** | **S.D.** | **Mean** | **S.D.** |
| **Circumference (mm)** | **C1** | 60.17 | 5.21 | 60.23 | 4.96 | 59.81 | 5.04 |
|  | **C2** | 47.38 | 3.92 | 47.62 | 3.78 | 47.42 | 4.08 |
|  | **C3** | 47.74 | 4.08 | 48.07 | 4.08 | 47.92 | 4.43 |
|  | **C4** | 44.67 | 3.86 | 44.78 | 3.70 | 44.98 | 4.21 |
|  | **C5** | 41.16 | 4.09 | 41.92 | 3.79 | 41.15 | 3.34 |
|  | **C6** | 56.75 | 4.15 | 56.66 | 3.72 | 56.74 | 4.17 |
|  | **C7** | 58.19 | 3.96 | 58.48 | 4.02 | 58.13 | 4.64 |
|  | **C8** | 55.07 | 3.74 | 55.23 | 3.88 | 55.14 | 4.18 |
|  | **C9** | 48.78 | 4.47 | 49.34 | 4.11 | 48.70 | 3.52 |
|  | **C10** | 68.68 | 6.67 | 68.31 | 6.40 | 68.86 | 6.46 |
|  | **C11** | 63.66 | 4.95 | 63.21 | 4.71 | 63.60 | 4.71 |
|  | **C12** | 61.36 | 4.12 | 61.77 | 4.51 | 61.70 | 4.67 |
|  | **C13** | 59.33 | 3.99 | 58.98 | 4.45 | 59.97 | 4.19 |
|  | **C14** | 55.17 | 4.58 | 55.29 | 4.95 | 56.24 | 4.30 |
|  | **C15** | 134.18 | 11.90 | 134.64 | 13.93 | 135.27 | 12.58 |
|  | **C16** | 190.91 | 13.95 | 194.00 | 13.57 | 191.37 | 13.99 |
|  | **C17** | 157.03 | 9.32 | 156.78 | 9.79 | 159.13 | 9.57 |
| **Length - palm (mm)** | **L1** | 51.39 | 4.23 | 51.33 | 4.19 | 56.02 | 4.50 |
|  | **L2** | 66.95 | 4.57 | 64.79 | 4.25 | 72.50 | 5.24 |
|  | **L3** | 74.83 | 4.97 | 71.84 | 4.32 | 81.05 | 5.25 |
|  | **L4** | 69.68 | 4.79 | 67.63 | 4.41 | 75.09 | 4.95 |
|  | **L5** | 56.31 | 4.98 | 54.09 | 4.69 | 61.06 | 4.98 |
|  | **L6** | 126.87 | 8.96 | 121.90 | 8.40 | 135.99 | 8.97 |
|  | **L7** | 171.97 | 10.59 | 171.65 | 10.30 | 179.95 | 11.06 |
|  | **L8** | 180.33 | 10.68 | 176.88 | 10.60 | 188.14 | 11.12 |
|  | **L9** | 168.94 | 10.93 | 164.56 | 10.42 | 177.17 | 11.36 |
|  | **L10** | 145.13 | 10.72 | 136.20 | 11.04 | 152.40 | 11.13 |
|  | **L11** | 105.79 | 7.40 | 105.17 | 7.72 | 107.31 | 7.51 |
|  | **L12** | 105.39 | 9.87 | 109.73 | 10.38 | 103.72 | 9.76 |
| **Length - dorsal (mm)** | **L13** | 55.94 | 6.01 | 56.39 | 6.26 | 51.84 | 5.26 |
|  | **L14** | 78.88 | 5.46 | 79.64 | 4.82 | 73.13 | 4.41 |
|  | **L15** | 88.08 | 5.78 | 88.15 | 5.53 | 82.17 | 5.58 |
|  | **L16** | 81.16 | 5.23 | 82.28 | 6.14 | 75.84 | 5.54 |
|  | **L17** | 64.33 | 6.35 | 64.21 | 6.37 | 59.12 | 5.30 |
|  | **L18** | 137.88 | 10.74 | 144.62 | 10.55 | 130.48 | 9.66 |
|  | **L19** | 171.82 | 11.75 | 172.31 | 11.06 | 166.83 | 11.37 |
|  | **L20** | 179.61 | 12.76 | 178.71 | 11.69 | 174.09 | 11.33 |
|  | **L21** | 170.57 | 12.57 | 171.09 | 12.13 | 164.97 | 11.44 |
|  | **L22** | 151.27 | 12.81 | 155.10 | 12.29 | 145.41 | 11.22 |
|  | **L23** | 17.60 | 4.78 | 18.25 | 5.11 | 17.14 | 4.86 |
|  | **L24** | 18.82 | 5.58 | 18.69 | 5.59 | 18.02 | 5.47 |
|  | **L25** | 18.93 | 6.06 | 18.97 | 5.76 | 18.39 | 5.69 |
|  | **L26** | 17.87 | 5.38 | 19.71 | 5.79 | 16.24 | 4.61 |
| **Length - web space (mm)** | **L27** | 13.78 | 2.48 | 13.87 | 2.74 | 13.25 | 2.63 |
|  | **L28** | 12.86 | 2.68 | 12.97 | 2.48 | 13.06 | 2.09 |
|  | **L29** | 12.11 | 2.11 | 12.14 | 2.50 | 13.04 | 2.74 |
|  | **L30** | 11.73 | 2.42 | 12.02 | 2.18 | 11.81 | 2.20 |
| **Angle (degree)** | **A1** | 80.40 | 13.05 | 87.82 | 7.58 | 88.88 | 12.02 |
|  | **A2** | 59.45 | 10.78 | 60.46 | 8.39 | 60.60 | 7.48 |
|  | **A3** | 48.85 | 7.39 | 51.59 | 7.94 | 54.12 | 6.20 |
|  | **A4** | 61.83 | 9.17 | 67.49 | 10.85 | 69.03 | 8.88 |
|  | **A5** | 63.47 | 9.19 | 60.94 | 7.46 | 61.69 | 7.46 |
|  | **A6** | 59.65 | 9.33 | 70.31 | 7.90 | 50.16 | 7.43 |
|  | **A7** | 49.97 | 10.63 | 58.85 | 11.05 | 43.04 | 7.86 |
|  | **A8** | 58.48 | 9.50 | 74.21 | 14.30 | 46.21 | 9.36 |
| **Surface area (mm^2^)** | **S1** | 2196.32 | 685.29 | 2335.01 | 678.22 | 2131.45 | 659.97 |
|  | **S2** | 7074.41 | 1045.89 | 7458.19 | 1011.89 | 7059.66 | 1060.14 |

^a^ Higher value are shaded grey
