# Supplementary material for: Impact of postural variation on hand measurements: Three-dimensional anatomical analysis
Source: PLoS One. 2021 Apr 23;16(4):e0250428. doi: 10.1371/journal.pone.0250428 (PMC8064611; doi:10.1371/journal.pone.0250428)
Supplement: S1 File — (DOCX) [file pone.0250428.s004.docx]

**Laboratory Protocol**

**Article title**

- Impact of postural variation on hand measurements: three-dimensional anatomical analysis

**Introduction**

- The impact of postural variations on hand anthropometry and distribution of skin strain ratios are important for the improvement of gloves fit, comfort, performance and functionality. This protocol helps to obtain the detailed hand dimension measurement at 3 different hand postures by 3D scanning method. 57 measurements, including 17 circumferential dimensions, 30 length dimensions, 8 angular dimensions and 2 surface area dimensions can be obtained.

**Materials**

- **Full-color handheld 3D scanner**
- Small flat colored landmarks

**Inclusion Criteria**

**Participants can be included in this study if they:**

- Aged between 18 and 40

AND

- No history of hand injuries

**Exclusion Criteria**

**Participants cannot be included in this study if:**

- have any hand musculoskeletal disorder or trauma

**Procedures**

1. *Participants were informed and provided written consent.*
2. *Participants were interviewed to obtain their demographic information.*
3. *Landmarks were* ***adhered to the hands of each subject (Fig 1).***
4. *3D scanning was carried out in bare hand condition with the use of Artec Eva handheld 3D scanner (Artec 3D, Luxembourg).*
5. *Each participant underwent three different posture conditions, namely relaxed, ball grip, and splayed in a randomized sequence.*
6. *The scanning process requires 30 seconds for each scan. Participants were asked to rest their elbow in a specially designed device.*
7. *Immediate image inspection was performed by the operator. Rescan if necessary.*
8. *Detailed hand measurements were recorded (Table 1).*


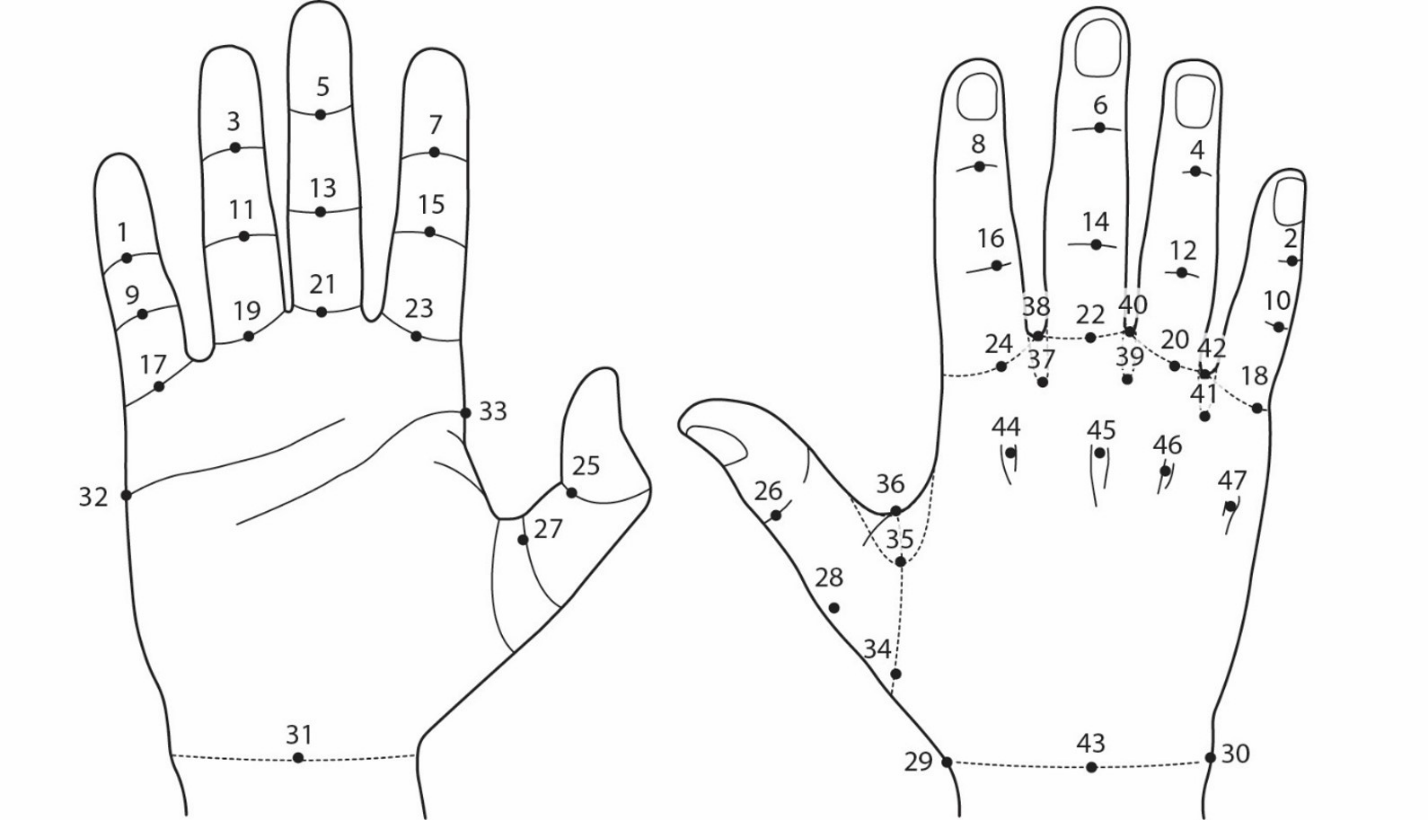


**Fig 1. Landmarks of hand**

**Table 1. Hand measurements**

| **Category** | **No.** | **Dimension** | **Landmarks** |
| --- | --- | --- | --- |
| **Circumference** | **C1** | IP joint of D1 | 25-26-25 |
|  | **C2** | Distal IP joint of D2 | 7-8-7 |
|  | **C3** | Distal IP joint of D3 | 5-6-5 |
|  | **C4** | Distal IP joint of D4 | 3-4-3 |
|  | **C5** | Distal IP joint of D5 | 1-2-1 |
|  | **C6** | Proximal IP joint of D2 | 15-16-15 |
|  | **C7** | Proximal IP joint of D3 | 13-14-13 |
|  | **C8** | Proximal IP joint of D4 | 11-12-11 |
|  | **C9** | Proximal IP joint of D5 | 9-10-9 |
|  | **C10** | Finger root of D1 | 27-28-27 |
|  | **C11** | Finger root of D2 | 23-24-23 |
|  | **C12** | Finger root of D3 | 21-22-21 |
|  | **C13** | Finger root of D4 | 19-20-19 |
|  | **C14** | Finger root of D5 | 17-18-17 |
|  | **C15** | Circumference between thumb and palm | 34-35-36-34 |
|  | **C16** | Hand circumference | 32-33-32 |
|  | **C17** | Wrist circumference | 29-30-31-29 |
| **Length - palm** | **L1** | Finger length of D1 | Tip of D1-27 |
|  | **L2** | Finger length of D2 | Tip of D2-23 |
|  | **L3** | Finger length of D3 | Tip of D3-21 |
|  | **L4** | Finger length of D4 | Tip of D4-19 |
|  | **L5** | Finger length of D5 | Tip of D5-17 |
|  | **L6** | Length from tip of D1 to wrist-crease | Tip of D1-31 |
|  | **L7** | Length from tip of D2 to wrist-crease | Tip of D2-31 |
|  | **L8** | Length from tip of D3 to wrist-crease | Tip of D3-31 |
|  | **L9** | Length from tip of D4 to wrist-crease | Tip of D4 -31 |
|  | **L10** | Length from tip of D5 to wrist-crease | Tip of D5 -31 |
|  | **L11** | Palm length | 21-31 |
|  | **L12** | Hand breadth | 32-33 |
| **Length - dorsal** | **L13** | Finger length of D1 | Tip of D1-28 |
|  | **L14** | Finger length of D2 | Tip of D2-24 |
|  | **L15** | Finger length of D3 | Tip of D3-22 |
|  | **L16** | Finger length of D4 | Tip of D4-20 |
|  | **L17** | Finger length of D5 | Tip of D5-18 |
|  | **L18** | Length from tip of D1 to wrist-crease | Tip of D1-43 |
|  | **L19** | Length from tip of D2 to wrist-crease | Tip of D2-43 |
|  | **L20** | Length from tip of D3 to wrist-crease | Tip of D3-43 |
|  | **L21** | Length from tip of D4 to wrist-crease | Tip of D4-43 |
|  | **L22** | Length from tip of D5 to wrist-crease | Tip of D5-43 |
|  | **L23** | Finger root of D2 to MCP joint | 24-44 |
|  | **L24** | Finger root of D3 to MCP joint | 22-45 |
|  | **L25** | Finger root of D4 to MCP joint | 20-46 |
|  | **L26** | Finger root of D5 to MCP joint | 18-47 |
| **Length - web space** | **L27** | Length between D1 and D2 | 35-36 |
|  | **L28** | Length between D2 and D3 | 37-38 |
|  | **L29** | Length between D3 and D4 | 39-40 |
|  | **L30** | Length between D4 and D5 | 41-42 |
| **Angle** | **A1** | Web space angle between D1 and D2 | 26-35-16 |
|  | **A2** | Web space angle between D2 and D3 | 16-37-14 |
|  | **A3** | Web space angle between D3 and D4 | 14-39-12 |
|  | **A4** | Web space angle between D4 and D5 | 12-41-10 |
|  | **A5** | Slant of web space between D1 and D2 | 35-36-31 |
|  | **A6** | Slant of web space between D2 and D3 | 37-38-31 |
|  | **A7** | Slant of web space between D3 and D4 | 39-40-31 |
|  | **A8** | Slant of web space between D4 and D5 | 41-42-31 |
| **Surface area** | **S1** | Finger root to MCP joint | Across 44-47 |
|  | **S2** | MCP joint to wrist line | 33-32-30-29-28-33 |

^a^ D: digit; IP: interphalangeal; and MCP: metacarpophalangeal
